# Is There a Link between Bisphenol A (BPA), a Key Endocrine Disruptor, and the Risk for SARS-CoV-2 Infection and Severe COVID-19?

**DOI:** 10.3390/jcm9103296

**Published:** 2020-10-14

**Authors:** Aeman Zahra, Cristina Sisu, Elisabete Silva, Sophie-Christine De Aguiar Greca, Harpal S. Randeva, Kamaljit Chatha, Ioannis Kyrou, Emmanouil Karteris

**Affiliations:** 1 Biosciences, College of Health, Medicine and Life Sciences, Brunel University London, Uxbridge UB8 3PH, UK; aeman.zahra@brunel.ac.uk (A.Z.); cristina.sisu@brunel.ac.uk (C.S.); elisabete.silva@brunel.ac.uk (E.S.); sophieja3@gmail.com (S.-C.D.A.G.); 2 Warwickshire Institute for the Study of Diabetes, Endocrinology and Metabolism (WISDEM), University Hospitals Coventry and Warwickshire NHS Trust, Coventry CV2 2DX, UK; harpal.randeva@uhcw.nhs.uk (H.S.R.); i.kyrou@aston.ac.uk (I.K.); 3 Aston Medical Research Institute, Aston Medical School, Aston University, Birmingham B4 7ET, UK; 4 Warwick Medical School, University of Warwick, Coventry CV4 7AL, UK; kamaljit.chatha@uhcw.nhs.uk; 5 Department of Biochemistry and Immunology, University Hospitals Coventry and Warwickshire NHS Trust, Coventry CV2 2DX, UK

**Keywords:** SARS-CoV-2, COVID-19, BPA, oestrogen receptors, ACE2, TMPRSS2, endocrine disruptors

## Abstract

Infection by the severe acute respiratory syndrome (SARS) coronavirus-2 (SARS-CoV-2) is the causative agent of a new disease (COVID-19). The risk of severe COVID-19 is increased by certain underlying comorbidities, including asthma, cancer, cardiovascular disease, hypertension, diabetes, and obesity. Notably, exposure to hormonally active chemicals called endocrine-disrupting chemicals (EDCs) can promote such cardio-metabolic diseases, endocrine-related cancers, and immune system dysregulation and thus, may also be linked to higher risk of severe COVID-19. Bisphenol A (BPA) is among the most common EDCs and exerts its effects via receptors which are widely distributed in human tissues, including nuclear oestrogen receptors (ERα and ERβ), membrane-bound oestrogen receptor (G protein-coupled receptor 30; GPR30), and human nuclear receptor oestrogen-related receptor gamma. As such, this paper focuses on the potential role of BPA in promoting comorbidities associated with severe COVID-19, as well as on potential BPA-induced effects on key SARS-CoV-2 infection mediators, such as angiotensin-converting enzyme 2 (ACE2) and transmembrane serine protease 2 (TMPRSS2). Interestingly, GPR30 appears to exhibit greater co-localisation with TMPRSS2 in key tissues like lung and prostate, suggesting that BPA exposure may impact on the local expression of these SARS-CoV-2 infection mediators. Overall, the potential role of BPA on the risk and severity of COVID-19 merits further investigation.

## 1. Introduction

Infection by the novel severe acute respiratory syndrome (SARS) coronavirus-2 (SARS-CoV-2) causes a severe new disease, i.e., COVID-19. Following the initial outbreak of COVID-19 cases at the end of 2019, COVID-19 reached pandemic status within months [1]. Growing data indicate that certain underlying diseases/conditions exhibit a direct association with significantly higher risk for adverse clinical outcomes of COVID-19 [1]. Indeed, chronic respiratory diseases (e.g., asthma and chronic obstructive pulmonary disease), cardiovascular disease (CVD), hypertension, diabetes, immunosuppression, and cancer are among the identified comorbidities which predispose individuals to severe COVID-19 [1].

Endocrine-disrupting chemicals (EDCs) are exogenous substances which can disrupt normal functions of the endocrine system in animals and humans, increasing the risk of adverse health effects [2]. Common EDCs include industrial solvents or lubricants and their by-products, pesticides, fungicides, plasticisers (e.g., bisphenol A (BPA) and phthalates), and pharmaceuticals [3]. EDCs are widespread in the environment and can accumulate across the entire food chain due to the long half-lives which commonly characterize these lipophilic chemicals, as well as the inability of the body to metabolize them [4]. Data from the US Centers for Disease Control and Prevention (CDC) suggest that humans can be exposed to hundreds of chemicals including EDCs [3]. Of note, research has suggested that increased and/or prolonged exposure of humans to EDCs can cause cardio-metabolic dysfunction, disorders of the reproductive system, endocrine-related cancers, and immune system dysregulation [5].

As more data on COVID-19 become available, the identified number of relevant predisposing risk factors is increasing, including factors such as obesity [6] and low socioeconomic and/or Black, Asian, and minority ethnic (BAME) background [7], which may be also linked to higher exposure to EDCs [8,9]. Indeed, a recent review has further proposed that long-term exposure to chemicals in mixtures, as well as lifestyle habits, may be linked to compromised immunity and predispose to the complications observed in patients with severe COVID-19 [10]. Moreover, a computational systems biology approach revealed that a number of signalling pathways which are dysregulated by EDCs (e.g., Th17 and advanced glycation end-products (AGE)/receptor for AGE (RAGE), AGE/RAGE, pathways) might also be related to the severity of COVID-19 [11]. As these detrimental effects of EDCs overlap with key risk factors for severe COVID-19, the hypothesis that exposure to EDCs may be also linked to the severity of COVID-19 merits further investigation [12].

Among the various EDCs, BPA is extensively used in a variety of products, including plastics, thermal receipts, and the lining of aluminium cans [13]. Accordingly, BPA is now one of the most frequently detected pollutants in the environment [14]. As such, in the present paper, we focus on the potential role of BPA in promoting the development of comorbidities which increase the risk of severe COVID-19, as well as on potential BPA-induced effects on key molecular targets which mediate the infection by SARS-CoV-2.

## 2. BPA and Comorbidities Predisposing to Severe COVID-19

### 2.1. BPA and Cardiometabolic Diseases

BPA is now recognized as a potential additional factor implicated in the development of cardio-metabolic diseases [15]. Indeed, BPA accumulates in adipose tissue and increases the number and size of adipocytes, thus contributing to increased adiposity and weight gain [16]. Moreover, a recent systematic review with a meta-analysis of the relevant epidemiological evidence reported that BPA exposure shows a significant positive association with indices of both generalized and central/abdominal obesity [17,18]. Similarly, systematic review data also support a relationship between BPA exposure and type 2 diabetes (T2DM) [19]. BPA exposure might be also associated with adiposity both in childhood and later in life [20]. Furthermore, a positive association has also been documented between low-dose BPA exposure during critical developmental periods (e.g., during foetal development) and metabolic diseases, such as T2DM [21].

Data from epidemiological and mechanistic studies also suggest a link between increased BPA exposure and hypertension [22], which is a key component of the metabolic syndrome and a leading CVD risk factor globally [23,24]. Of note, this positive association was documented in a multi-ethnic sample of US adults, independently of confounding factors such as age, gender, smoking, body mass index (BMI), diabetes, and cholesterol levels [25]. A positive association was noted between urinary BPA levels and hypertension in 1380 subjects from the National Health and Nutritional Examination Survey (NHANES), independent of confounding factors such as age, gender, race/ethnicity, diabetes, smoking, BMI, and total serum cholesterol levels [25]. This was further corroborated by another study of 2588 sera samples from the Thai NHANES, where BPA exhibited a positive association with hypertension which was also independent of age, sex, BMI, diabetes, and oestrogen levels [26]. Finally, in a more recent study in Seoul where 560 elderly participants were recruited, BPA exposure was associated with increased blood pressure and decreased heart rate variability, which are both risk factors of CVD [27]. Moreover, in terms of underlying mechanisms, a number of studies point towards an involvement of BPA in vascular dysfunction. For example, in the population-based Prospective Investigation of the Vasculature in Uppsala Seniors study, BPA was related to the echogenicity of atherosclerotic plaques of the carotid arteries, suggesting a role for plaque-associated chemicals in atherosclerosis [28]. In addition, high BPA serum levels were also associated with increased carotid intima-media thickness in a cross-sectional study of adolescents and young adults [29]. In line with these findings, in an in vivo study where BPA was administered in male rats, BPA was shown to exert a cardiotoxic effect, inducing a state of oxidative stress and leading to the overproduction of free radicals [30]. Furthermore, in a more recent study using cardiomyoblasts in vitro, BPA induced pro-inflammatory interleukins (IL) involved in CVD (i.e., IL-8, IL-6, and IL-1β), whilst also enhanced doxorubicin-induced cardiotoxicity phenomena [31].

Finally, a strong relationship between BPA and circulating androgen levels has been shown, suggesting a link to ovarian dysfunction and polycystic ovary syndrome (PCOS) [32]. The latter is also strongly linked to the metabolic syndrome in women [33,34], with systematic review data suggesting that BPA is involved in both hyperandrogenism and insulin resistance of PCOS [35,36].

Overall, it is noteworthy that CVD and all these chronic diseases which commonly cluster within the metabolic syndrome spectrum (e.g., obesity, T2DM, and hypertension) are now consistently recognized as key factors that predispose to severe COVID-19 [37,38,39,40,41,42]. Thus, BPA exposure by promoting the development of these cardio-metabolic diseases over time may be also indirectly linked to higher risk of severe COVID-19, particularly in older individuals that are at a high risk group for severe COVID-19 [43].

### 2.2. BPA and Cancer

BPA exposure has been linked to carcinogenicity, especially of hormone-dependent tumours, such as prostate, breast, and ovarian cancers [44]. As such, prenatal BPA exposure may influence the development of prostate cancer in later life, and also increase the frequency of breast tumours through either alteration of foetal glands or by mediating oestrogen-dependent growth of tumour cells [16]. Interestingly, pregnant mice which were exposed to BPA levels within the range of human exposure showed increased prostate volume and decreased sperm production in the adult male offspring [45,46,47]. Furthermore, increasing evidence from both in vitro and animal studies suggest that BPA exposure, even at low doses, may have carcinogenic effects on breast cancer [48]. Moreover, BPA appears to increase the risk of endometriosis which, in turn, increases the risk of both coronary heart disease and ovarian cancer [49,50]. Finally, BPA exposure may induce endometrial stromal cell invasion and has a positive association with peritoneal endometriosis [51].

To date, an increasing body of evidence, including meta-analysis data, indicate that cancer comorbidity exhibits an association with both the risk and severity of COVID-19 [52]. In a recent UK study of 156 cancer patients with confirmed COVID-19 diagnosis it was shown that patients who live longer with cancer are at greater risk of infection as well as of COVID-19 related death [53]. Of note, cancer patients with urological/gynaecological, breast, and lung cancers, as well as haematological malignancies, were presented with severe COVID-19 [53]. As aforementioned before, BPA has been involved in the development of certain cancers and a number of mechanisms have been proposed. For example, exposure of mouse mammary tumor virus (MMTV)-erbB2 mice to low BPA doses in utero has been shown to lead in mammary tumourigenesis and mammary epithelial reprogramming involving the oestrogen receptor (ER)-erbB2 pathway [54]. Similarly, perinatal exposure of adult CD-1 mice to BPA resulted in induction of mammary intraductal hyperplasia [55]. Furthermore, in an in vitro study, BPA increased the migration and invasion of triple-negative breast cancer cells, while it also induced protein expression of matrix metalloproteinase-2 (MMP-2) and MMP-9 [56].

However, a systematic review reporting on the effects of cancer—among other comorbidities—on COVID-19 severity concluded that this association must be interpreted with caution due to a number of confounding factors, including old age, smoking history, and co-existing comorbidities of the involved study participants, as well as the sample size of these studies [42]. Accordingly, additional research should also be focused on the potential links between endocrine-dependent tumours with known associations to BPA exposure (e.g., prostate, breast, and ovarian cancers) and COVID-19, including exploring the potential underlying molecular mechanisms using in vitro and in vivo models, as well as clinicopathological data.

### 2.3. BPA and Modulation of Immune System Responses

An increasing number of studies have also drawn attention to the potential involvement of BPA in modulating immune system responses, and, particularly, to its potential ability to facilitate airway inflammation and respiratory allergies, as well as impair immunotolerance to dietary proteins [57,58,59,60]. Multiple mechanisms have been suggested to mediate the potential effects of BPA on the immune system, such as direct effects on relevant receptors (e.g., oestrogen receptors) and cellular signalling pathways, as well as epigenetic effects and changes of the gut microbiome [57]. Overall, BPA exposure may impact on both the sub-type and function of the adaptive and innate immune system cells, leading to changes in produced cytokines and chemokines (e.g., upregulation of pro-inflammatory cytokines such as interferon-gamma (IFN-γ), tumor necrosis factor alpha (TNF-α), IL-10, and IL-4) and decreased T regulatory (Treg) cells [57,58]. Interestingly, oral BPA exposure of ovariectomized rats has been shown to induce a pro-inflammatory response in their adult female offspring, suggesting potential long-term effects of BPA on the immune system of the progeny [61].

In this context, it should be highlighted that COVID-19 severity also appears to be linked to increased local and systemic levels of an array of pro-inflammatory cytokines and chemokines (e.g., TNF-α, IL-1β, IL-6, IL-8, and IL-2) [62,63,64]. This may further induce a vicious cycle of hyperinflammatory reactions in certain patients with severe COVID-19, resulting in an underlying cytokine storm with adverse clinical outcomes [62,63,64]. As these pro-inflammatory pathways may be also triggered by increased and/or prolonged exposure to BPA, this may represent an additional indirect pathophysiologic mechanism via which BPA could potentially increase the risk of severe COVID-19 in vulnerable individuals, particularly those with T2DM, obesity, hypertension, and CVD who already exhibit various degrees of underlying low-grade chronic inflammation [62]. However, recently it was shown that critically ill patients with COVID-19 suffering with acute respiratory distress syndrome (ARDS) had lower circulating cytokine levels when compared with sepsis or other critically ill patients [65]. This was further corroborated by data demonstrating that although COVID-19 patients exhibited increased pro-inflammatory cytokine levels (e.g., IL-16, IL-10, and monocyte chemoattractant protein-1, MCP-1), these levels were not as high as in other non-COVID-19 patients suffering from cytokine-release syndrome [66]. Therefore, it appears that there might be a higher order of complexity regarding the role and potential implications of an underlying “cytokine storm” in COVID-19 that also merits further investigation. In this context, the role of BPA on immunity should be further investigated as this may be further implicated in the potential mechanisms linking BPA with higher risk for COVID-19 [57].

### 2.4. BPA and Links to Pregnancy and Placentation Complications

A growing body of evidence has further shown that BPA exposure, even at low doses, may have adverse effects on the outcomes of pregnancy in humans, resulting in potentially harmful conditions for both the mother and the offspring (e.g., affecting the normal development of the foetus and/or causing problems later in life) [67,68,69,70,71,72,73]. There is also a correlation between BPA exposure and preeclampsia during pregnancy [74,75], which is characterized by newly diagnosed hypertension and proteinuria [76] and is associated with increased risk of both maternal mortality and health problems for the offspring later in life (e.g., obesity and T2DM) [76,77].

Although more data are necessary to prove a direct association between BPA exposure and preeclampsia or placental alterations, the potential link between BPA and preeclampsia is of particular interest in relation to COVID-19, given that pregnant women with severe COVID-19 can develop a preeclampsia-like syndrome [78]. So far, single cases of COVID-19 causing preeclampsia or pregnancy-induced hypertension have been described [79,80]. Moreover, Shanes et al. found that third trimester placentas from women with COVID-19 had significantly higher probability of vascular malperfusion, showing features such as abnormal or injured maternal vessels or intervillous thrombi [81]. Similarly, Baergen et al. found that half of the studied placentas in a cohort of 20 mothers with COVID-19 showed evidence of foetal vascular thrombosis or foetal vascular malperfusion [82]. In another study, in five pregnant women with COVID-19 who delivered at term without complications, all five placentas showed focal avascular villi and thrombi in larger vessels [83], although no direct SARS-CoV-2 infection of the placenta was noted and the placental changes were attributed to systemic rather than local infection [83]. Given that, in addition to the pro-thrombotic nature of pregnancy, COVID-19 appears to be associated with pro-thrombotic effects on both the placenta [83] and systemic infection [84], importance has been given to continuing prophylactic aspirin in women with COVID-19 at risk for preeclampsia, although some studies have questioned whether non-steroidal anti-inflammatory drugs can exacerbate COVID-19 symptoms [85].

Overall, whether COVID-19 symptoms could be exacerbated in pregnant women and whether BPA exposure may further increase the relevant risk need further investigation, particularly since the immune system during pregnancy is in a state of constant adaptation with pregnant women being more susceptible to respiratory infections [79]. Notably, a study from Spain on the clinical outcomes of 60 pregnant women with confirmed COVID-19 has reported that most of these patients had a good clinical outcome, with one-third developing pneumonia and 5% classified as being in critical condition [86]. Similar findings were reported by another recent study showing that there were no severe cases of pneumonia and no maternal deaths in pregnant women with COVID-19 [87]. So far, there is very limited evidence on the potential vertical transmission of COVID-19 from a mother to a child, with a recent review of the relevant existing literature reporting little evidence for such transmission [88]. However, there are rare reported cases of vertical transmission of COVID-19 from mothers to neonates. For example, two cases of COVID-19 (one delivered vaginally after spontaneous labour and one via caesarean section) were found in the neonates of a cohort of 22 women who were affected by COVID-19 during the third trimester of pregnancy [89]. Although such research studies on pregnancy and COVID-19 are increasing, currently there are no reported studies on BPA blood/urine levels in pregnant women diagnosed with COVID-19 and their offspring.

## 3. BPA and Key Molecular Targets of SARS-CoV-2

SARS-CoV-2 infection of target/host cells is mediated by a number of cellular receptors and proteases. As such, SARS-CoV-2 binds with high affinity to angiotensin-converting enzyme 2 (ACE2) on the cell membrane, which facilitates viral entry into host cells [90]. Moreover, transmembrane serine protease 2 (TMPRSS2) is co-expressed with ACE2 on the cell membrane and it can prime the viral spike proteins, thus mediating the fusion of the virus with the membrane lipid layer and its uptake into host cells [91]. In addition, cathepsin L (CTSL), a lysosomal protease which is known to mediate the cellular entry of the SARS virus via endosomes by priming the viral spike proteins for membrane fusion [92], appears to also facilitate the infection of host cells by SARS-CoV-2 [91]. Similarly, furin is a protease known for cleaving inactive precursor proteins into their biologically active products [93], while furin inhibitors have been investigated in the search for novel SARS-CoV-2 treatments since a relevant site has been discovered in the protein sequence of the SARS-CoV-2 spike protein [94,95].

As more research is now focused on the role of cellular mediators in SARS-CoV-2 infection and potential factors affecting their expression/functions, we also present data on the potential effects of BPA on these key SARS-CoV-2 infection mediators in this review.

### 3.1. BPA and Expression of TMPRSS2

Evidence from animal studies indicate that BPA can affect TMPRSS2 expression. Indeed, when BPA was administered subcutaneously to male rats from days 1 to 3, the expression of TMPRSS2 was upregulated in their medial amygdala [96]. This BPA-induced increase in the density of TMPRSS2 immunoreactive cells in the medial amygdala of neonatal male rats suggests that BPA has the potential to disturb central nervous system (CNS) and neurodevelopmental processes [96]. Interestingly, increasing attention is now placed on the neurotropism of coronaviruses, such as SARS-CoV-2, and their potential effects on neuropathogenesis and the CNS [97,98].

On the other hand, in vitro studies in Ishikawa cells, i.e., a well-characterized human endometrial cell line which can be used as an in vitro model for testing potential estrogenic effects of various chemicals, showed that BPA treatment can induce the downregulation of TMPRSS2 [99]. Moreover, we have recently published our research findings on the effects of physiologically relevant doses of BPA on the human placenta using non-syncytialised and syncytialised BeWo cells as in vitro models [100]. In the context of COVID-19, we revisited the microarray data from these experiments and we found that the applied BPA treatment induced a modest increase of TMPRSS2 expression in non-syncytialised and syncytialised BeWo cells, with no effect on ACE2 and CTSL expression (unpublished data). Interestingly, one of the significantly enriched processes in non-syncytialised BeWo cells treated with BPA (3 nM) in our experiments appears to be implicated in the regulation of viral life cell cycle [100].

Considering the available evidence which suggests that BPA can variably impact on the expression of TMPRSS2, further research is needed in order to explore whether any such BPA-induced effects on this key SARS-CoV-2 infection mediator may have a clinically relevant impact on the risk of developing COVID-19 and its subsequent severity.

### 3.2. BPA and Expression of ACE2 and Furin

Limited data on the potential effects of BPA on the expression of ACE2 and furin exist so far. As BPA is suspected to promote male reproductive impairments, an ex vivo toxicogenomic study using a rat seminiferous tubule culture model to investigate BPA effects on spermatogenesis showed that exposure to low-dose BPA (1 nM) can downregulate ACE2 and furin after 21 and 14 days of exposure, respectively [101]. Furthermore, a study with RNA-seq analyses of the testicular mRNA libraries of adult male rare minnows (*Gobiocypris rarus*; a small cyprinid fish used as a model for aquatic toxicology research) which were exposed to different BPA concentrations (1, 15, and 225 μg/L for 7 days) showed that ACE2, which is expressed in Leydig cells and may serve as a regulator of testicular steroidogenesis, was one of the most significantly increased genes of the renin-angiotensin system following BPA exposure (1 μg/L for 7 days) [102]. On the other hand, another study investigating the potential adverse impact of BPA exposure (50 mg/kg of body weight for 6 weeks) during puberty in male mice showed significantly decreased ACE2 protein expression in the cauda epididymis of BPA-exposed mice [103]. As men are consistently at higher age-adjusted risk for severe COVID-19 compared with women [104], and there is currently ongoing research regarding whether the human reproductive system constitutes an additional target for SARS-CoV-2 infection [105,106,107,108], future research studies should also investigate whether BPA may play a role in such COVID-19-related complications by modulating the local expression of key SARS-CoV-2 infection mediators, such as ACE2.

### 3.3. Co-expression of Receptors Mediating BPA Effects with SARS-CoV-2 Infection Mediators

**Figure 1:**
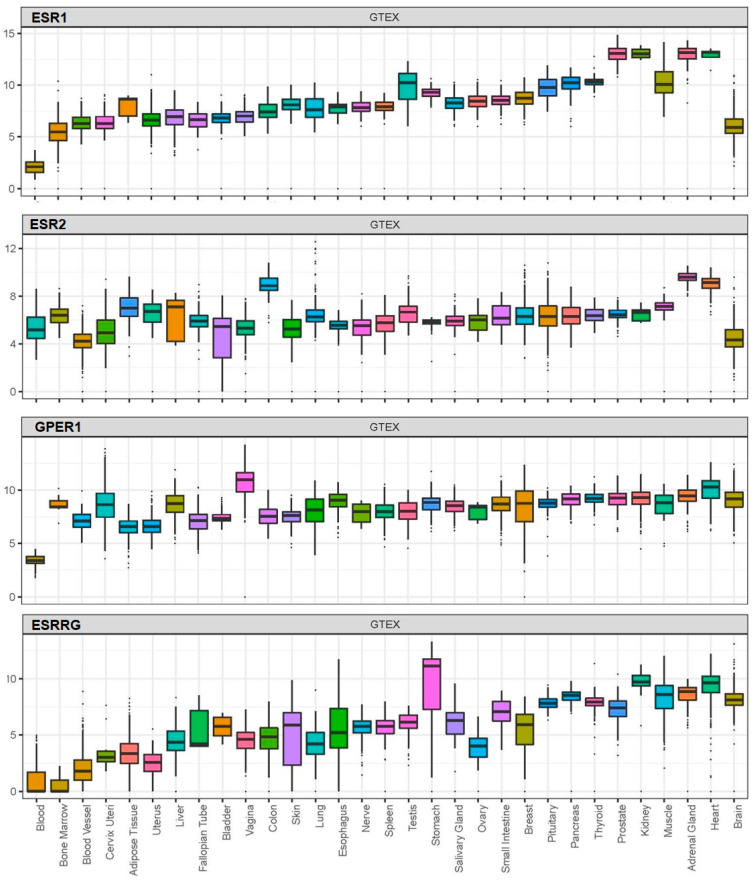
Expression (log2(norm_count+1)) of the nuclear oestrogen receptors ERα (ESR1) and ERβ (ESR2), G protein-coupled membrane-bound oestrogen receptor (GPR30 or GPER1), and oestrogen-related receptor gamma (ESRRG) across human tissues based on available data from the Genotype-Tissue Expression (GTEx) project.

BPA exerts its effects by acting on receptors which, based on available data from the Genotype-Tissue Expression (GTEx) project, are widely distributed in human tissues, including nuclear oestrogen receptors (ERα and ERβ), membrane-bound oestrogen receptor (G protein-coupled receptor 30; GPR30), and human nuclear receptor oestrogen-related receptor gamma (Figure 1) [100,109,110,111].

**Figure 2:**
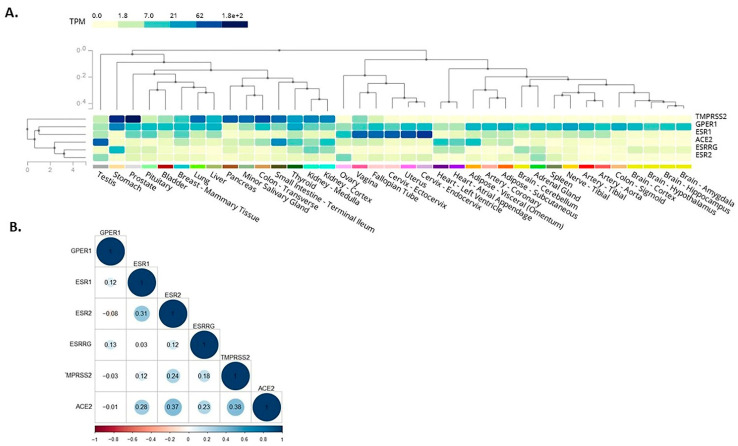
Co-expression (**A**) and correlation (**B**) of the main known receptors, i.e., nuclear oestrogen receptors ERα (ESR1) and ERβ (ESR2), membrane-bound oestrogen receptor (G protein-coupled receptor 30; GPR30 or GPER1), and oestrogen-related receptor gamma (ESRRG) which mediate the effects of bisphenol A (BPA) with key SARS-CoV-2 infection mediators, i.e., angiotensin-converting enzyme 2 (ACE2) and transmembrane serine protease 2 (TMPRSS2), based on available data from the Genotype-Tissue Expression (GTEx) project.

Here, we expanded on these in silico observations by assessing the co-expression of receptors mediating BPA effects with SARS-CoV-2 infection mediators. As such, among these receptors which mediate BPA effects, the membrane-bound oestrogen receptor GPR30 appeared to co-localise with TMPRSS2 in the lung, colon, stomach, small intestine, thyroid, kidney, liver, and prostate (Figure 2A). This finding suggests that BPA exposure may impact via GPR30 on these SARS-CoV-2 infection mediators in these tissues and, thus, have potential implications on the severity of COVID-19 (e.g., on the consequences of SARS-CoV-2 infection in the lungs). We have dissected these data further, using available data from the GTEx project, to investigate any potential correlation among the expression patterns of these genes. For this, we computed the Pearson correlation coefficient between the genes’ expression levels in healthy tissue samples. A high degree of correlation was noted between ACE2 with ERβ (0.37) and TMPRSS2 (0.38), whereas moderate correlation was noted between ACE2 with ERα (0.28) and oestrogen-related receptor gamma (0.23) (Figure 2B). The results suggest that these genes have a correlated expression pattern.

## 4. Conclusions

**Figure 3:**
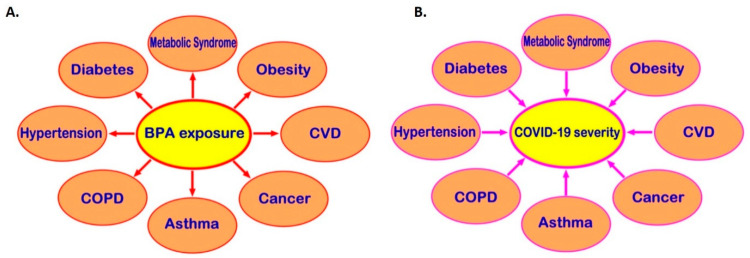
Potential links via which bisphenol A (BPA) could indirectly increase the risk for severe COVID-19. Exposure to BPA can promote the development of multiple cardio-metabolic diseases and endocrine-related cancers (**A**). These comorbidities predispose to worse COVID-19 clinical outcomes (**B**); hence, BPA exposure may be indirectly linked to higher risk of severe COVID-19. CVD: cardiovascular disease; COPD: chronic obstructive pulmonary disease; COVID-19: coronavirus disease 2019.

Exposure to BPA, one of the most common EDCs, can promote the development of cardio-metabolic diseases, endocrine-related cancers, and immune system dysregulation and, through that, may be indirectly linked to higher risk of severe COVID-19 (Figure 3). Moreover, receptors which directly mediate BPA effects, such as the membrane-bound oestrogen receptor GPR30, are widely distributed in human tissues and may co-localise with SARS-CoV-2 infection mediators (e.g., co-localisation of GPR30 with TMPRSS2 and CTSL in the lung), potentially affecting their local tissue expression. Therefore, it becomes evident that there might be potential implications of exposure to BPA and other common EDCs on the risk of SARS-CoV-2 infection and the severity of COVID-19 [11,12]. This is a developing topic and clearly further in vitro, computational, preclinical, and in vivo studies are needed to elucidate any such direct links between BPA and COVID-19 and clarify the molecular mechanisms that may be involved. Ultimately, this can lead to a new framework and guidelines for reducing relevant EDC exposure(s) in the context of COVID-19, particularly in high COVID-19 risk groups (e.g., men and older individuals, as well as patients with comorbidities such as T2DM, hypertension, obesity, and CVD).
